# Modulations of Homeostatic ACE2, CD147, GRP78 Pathways Correlate with Vascular and Endothelial Performance Markers during Pulmonary SARS-CoV-2 Infection

**DOI:** 10.3390/cells13050432

**Published:** 2024-02-29

**Authors:** Annuurun Nisa, Ranjeet Kumar, Santhamani Ramasamy, Afsal Kolloli, Judith Olejnik, Sallieu Jalloh, Suryaram Gummuluru, Selvakumar Subbian, Yuri Bushkin

**Affiliations:** 1 Public Health Research Institute, New Jersey Medical School, Rutgers University, Newark, NJ 07103, USA; annuurunnias@gmail.com (A.N.); rk879@njms.rutgers.edu (R.K.); santhuvet@gmail.com (S.R.); ak1482@njms.rutgers.edu (A.K.); 2 Department of Virology, Immunology & Microbiology, Boston University School of Medicine, Boston, MA 02130, USA; jolejnik@bu.edu (J.O.); cjalloh09@gmail.com (S.J.); rgummulu@bu.edu (S.G.); 3 National Emerging Infectious Diseases Laboratories, Boston University, Boston, MA 02218, USA

**Keywords:** COVID-19, ACE2, CD147, GRP78, single-molecule FISH, thrombosis, vascular dysfunction, hamster

## Abstract

The pathologic consequences of Coronavirus Disease-2019 (COVID-19) include elevated inflammation and dysregulated vascular functions associated with thrombosis. In general, disruption of vascular homeostasis and ensuing prothrombotic events are driven by activated platelets, monocytes, and macrophages, which form aggregates (thrombi) attached to the endothelium lining of vessel walls. However, molecular pathways underpinning the pathological interactions between myeloid cells and endothelium during COVID-19 remain undefined. Here, we tested the hypothesis that modulations in the expression of cellular receptors angiotensin-converting enzyme 2 (ACE2), CD147, and glucose-regulated protein 78 (GRP78), which are involved in homeostasis and endothelial performance, are the hallmark responses induced by SARS-CoV-2 infection. Cultured macrophages and lungs of hamster model systems were used to test this hypothesis. The results indicate that while macrophages and endothelial cells are less likely to support SARS-CoV-2 proliferation, these cells may readily respond to inflammatory stimuli generated by the infected lung epithelium. SARS-CoV-2 induced modulations of tested cellular receptors correlated with corresponding changes in the mRNA expression of coagulation cascade regulators and endothelial integrity components in infected hamster lungs. Among these markers, tissue factor (TF) had the best correlation for prothrombotic events during SARS-CoV-2 infection. Furthermore, the single-molecule fluorescence in situ hybridization (smFISH) method alone was sufficient to determine the peak and resolution phases of SARS-CoV-2 infection and enabled screening for cellular markers co-expressed with the virus. These findings suggest possible molecular pathways for exploration of novel drugs capable of blocking the prothrombotic shift events that exacerbate COVID-19 pathophysiology and control the disease.

## 1. Introduction

Severe acute respiratory syndrome coronavirus 2 (SARS-CoV-2) infects the respiratory tract and airway epithelial cells, causing coronavirus disease-2019 (COVID-19) [1,2]. SARS-CoV-2 primarily uses angiotensin-converting enzyme 2 (ACE2) as a receptor to infect and damage ciliated epithelial cells [3,4]. Moderate to severe COVID-19 cases are characterized by excessive inflammation in the lung, induced by increased production of proinflammatory cytokines and loss of a balanced immune regulation [5,6]. While molecular pathways underpinning hyper-inflammation remain undefined, RNA sensing of SARS-CoV-2 in the lung may prime local macrophages or epithelial cells and further exacerbate the local inflammatory environment [7].

COVID-19 causes progressive respiratory failure resulting from diffuse alveolar damage and systemic coagulopathy, thrombosis, and capillary inflammation, linking alveolar responses to endothelial dysfunction [8,9,10,11,12]. The endothelium regulates homeostasis and thrombosis in cooperation with hematopoietic cells that also express coagulation cascade key signaling molecules [13]. Activated by excessive inflammation or injury, endothelium produces and releases regulatory components such as tissue factor (TF) and von Willebrand factor (vWF), which promote aggregation of platelets and enhance control of fibrinolysis [14,15]. Activation of endothelium and ensuing inflammation shifts the vascular equilibrium from resting to a thrombotic state, which results in endothelial injury and disruption of intercellular junctions affecting numerous cell surface markers [8,16,17]. Accordingly, thrombotic complications and increased endothelial permeability are the hallmarks of COVID-19 pathophysiology. While endothelial and myeloid cells are less likely to support productive viral infection, these tissues may readily respond to proinflammatory stimuli generated by the infected lung epithelium [18,19].

The molecular mechanisms linking the prothrombotic shift and endothelial dysfunction have not been fully elucidated. The key causative factors driven by infection-induced inflammation are alterations in blood content, flow, and vessel endothelium [13,14]. We reasoned that since ACE2, CD147, and glucose-regulated protein 78 (GRP78) signaling pathways have crucial roles in homeostatic control and maintenance of endothelial performance and are broadly expressed in lung tissues including epithelium, endothelium and myeloid immune cells, their expression may be modulated by SARS-CoV-2 induced inflammation. In this trio of key regulatory molecules involved in homeostasis, the multi-functional roles of CD147 and GRP78 are better known, while the fundamental role of ACE2 also becomes evident [20,21]. A positive correlation between ACE2 activation and thrombotic activity has been demonstrated [22,23]. ACE2 is also a primary SARS-CoV-2 receptor [24,25]. In the lung, only 0.64% of cells express ACE2, most of them alveolar type II cells (AT2) [26]. Other cell types, including alveolar type I cells (AT1), airway epithelial cells, macrophages, fibroblasts, and endothelial cells, also express ACE2 but at lower levels than AT2 [26]. While signaling through ACE2 upon viral entry remains poorly defined, ACE2 expression and corresponding intracellular signaling may be downregulated, leading to increased vascular permeability and a prothrombotic phenotype in COVID-19 cases; a pathology that is exacerbated by certain comorbidities such as heart diseases [23,27,28]. The downregulation of ACE2 in lung tissues and plasma of patients with severe COVID-19 and its association with the prothrombotic phenotype has been hypothesized [23], (reviewed in [27]). Furthermore, the loss of ACE2 under conditions promoting acute lung injury (i.e., acid challenge or sepsis) increased vascular permeability in murine lungs [29]. Yet, this earlier evidence supporting downregulation of ACE2 during COVID-19 is challenged by recent findings that upregulated activity of ADAM17, a membrane-bound metalloproteinase, can cleave membrane ACE2, thus leading to upregulated systemic levels and activity of soluble ACE2 during SARS-CoV-2 infection [30,31]. Thus, RNAseq data obtained from the myocardial tissues of COVID-19 patients showed an association between the expression of ACE2, ADAM17, and other potential accessory proteases in pericytes and fibroblasts that might facilitate SARS-CoV-2 infection [32]. Nonetheless, plasma levels of ACE2 were found to be persistently elevated in patients during COVID-19, which might be due to enhanced proteolytic processing and shedding of ACE2 [33,34]. It was suggested that binding of SARS-CoV-2 to membrane-bound ACE2 causes downregulation of membrane ACE2 expression, which contributes to the dysregulation of the renin-angiotensin system (RAS) [33]. Interestingly, blocking of the ACE2-viral S protein complex degradation restored the surface expression of ACE2 and improved viral clearance in a hamster model of SARS-CoV-2 infection [35].

In addition to ACE2, the CD147 and GRP78 pathways may be involved in vascular disease mechanisms altered during COVID-19. CD147 is upregulated in inflammation in many tissues as well as in activated platelets. It promotes thrombosis through homotypic interactions with endothelium and monocytes and by recruiting monocytes to the vascular wall [36,37,38]. CD147 is a multi-functional receptor; it facilitates angiogenesis through interactions with vascular endothelial growth factor A (VEGF-A) and VEGF receptor type 2 (VEGFR-2) and activates the urokinase-type plasminogen/plasminogen activator inhibitor-1 (uPA/PAI-1) system creating conditions that promote a prothrombotic phenotype [37,38,39,40]. GRP78 functions as a chaperone for the folding, maturation, and transport of peptides in the endoplasmic reticulum (ER) [41]. Under stress conditions, GRP78 can dissociate from the ER molecular complexes and translocate to the cell surface, where it then functions as a signaling molecule regulating anti-apoptotic and promigratory pathways [41,42,43]. Surface GRP78 also has a role in thrombosis by binding to and inhibiting TF [41,44,45]. Additionally, GRP78 can regulate endothelial integrity [46]. Translocation of GRP78 to the cell surface is correlated with augmented O-glycosylation of VE-cadherin and the formation of GRP78/VE-cadherin surface complexes [46]. VE-cadherin also forms complexes with VEGF-A/VEGFR-2 molecules. Upregulation of surface GRP78/VEGFR-2 complexes lead to a loss of endothelial barrier integrity and facilitates the migration of monocytes through the endothelium [43,46].

These findings point to the possibility that ACE2, CD147 and GRP78 pathways are involved in the perturbation of homeostasis leading to vascular dysfunction that is characteristic of COVID-19. Thus, our objective in this study was to evaluate the RNA modulations of these key pathways in human macrophage in vitro models, PMA-differentiated THP-1 cells (THP-1/PMA), and primary monocyte-derived macrophages (MDMs) with induced constitutive expression of ACE2 enabling effective infection and restoring productive SARS-CoV-2 replication [18], and in a Golden Syrian hamster model, which recapitulates the pathophysiology of mild-to-moderate COVID-19 [47]. Data from our study suggest that SARS-CoV-2 infection-induced upregulation of prothrombotic signature molecules associated with modulations of ACE2, CD147, and GRP78 pathways are detectable in animal model systems. The prothrombotic shift contributes to endothelial dysfunction and increased vascular permeability in infected lungs that is emblematic of severe COVID-19 pathogenesis.

## 2. Materials and Methods

### 2.1. Cell Cultures

Vero E6 cells (ATCC CRL-1586, Manassas, MA, USA) were maintained in Dulbecco’s modified Eagle’s medium (DMEM) supplemented with 10% FBS and 100 μg/mL primocin. THP-1 cells (ATCC, TIB-202) were maintained in RPMI/1640 (ThermoFisher Scientific, Waltham, MA, USA) containing 10% FBS and 1% pen/strep (Sigma-Aldrich, St. Louis, MO, USA). To generate THP-1/ACE2^+^ cells, THP-1 cells were transduced with pLenti-ACE2-IRES-puro lentivector and cultured in puromycin-containing media (2 μg/mL). Cells with robust surface expression of ACE2 were sorted using a MoFlo cell sorter (Beckman Coulter, Bria, CA, USA) and cultured in puromycin-supplemented media. All cell lines were routinely tested for mycoplasma contamination and confirmed negative. For THP-1 monocyte to macrophage differentiation, THP-1 cells were stimulated with 100 nM PMA (Sigma-Aldrich, St. Louis, MO, USA) for 48 h. Human monocyte-derived macrophages (MDMs) were derived from CD14^+^ peripheral blood monocytes by culturing cells in RPMI/1640 media containing 10% heat-inactivated human AB serum (Sigma-Aldrich, St. Louis, MO, USA) and recombinant human M-CSF (20 ng per mL; Peprotech, Cranbury, NJ, USA) for 5–6 days. To generate MDMs expressing ACE2, cells were co-infected with ACE2-expressing lentiviruses (100 ng, based on p24^gag^ ELISA, per 1 × 10^6^ cells) and SIV3+ (Vpx-expressing) VLPs for 3 days. Surface expression of ACE2 in both THP-1 and MDMs was confirmed by flow cytometry, as previously described for stable expression of wild-type and mutant ACE2 and CD169 molecules [18].

### 2.2. Propagation of SARS-CoV-2 and In Vitro Infection

For in vitro infection studies, SARS-CoV-2 stocks (isolate USA_WA1/2020; kindly provided by CDC’s Principal Investigator Natalie Thornburg and the World Reference Center for Emerging Viruses and Arboviruses (WRCEVA)) were propagated in Vero E6 cells cultured in DMEM supplemented with 2% fetal FBS and 100 μg/mL primocin. To remove confounding cytokines and other factors, viral stocks were purified by ultracentrifugation through a 20% sucrose cushion at 80,000× *g* for 2 h at 4 °C [48]. The SARS-CoV-2 titer was determined in Vero E6 cells by the tissue culture infectious dose 50 (TCID_50_) assay using the Spearman–Kärber algorithm. For hamster infection studies, SARS-CoV-2 (strain USA-WA1/2020) was obtained from BEI Resources (ATCC, Manassas, VA, USA). Virus propagation and titration were determined by plaque-forming unit (PFU) assays using Vero E6 cells, as described previously [4,49]. For smFISH analysis, 1 × 10^6^ cells (THP-1/PMA or MDMs) were seeded in 6-well plates containing 3–4 coverslips per well in FBS-supplemented RPMI media. The next day, cells were infected with purified SARS-CoV-2 at the multiplicity of infection (MOI) 5. The virus inoculum was removed after 1 h of virus attachment, cells were washed twice with PBS, and fresh media was added. As a control for the in vitro studies, we used a LV-3′LTR lentivector expressing empty vector components as we reported previously [18]. At indicated time points, the cells were fixed in 10% neutral buffered formalin for at least 6 h at 4 °C and removed from the biosafety level 4 (BSL4) laboratory for staining and imaging analysis in accordance with approved standard operating procedures. All cell culture experiments with SARS-CoV-2 were performed in the BSL4 facility of the National Emerging Infectious Diseases Laboratories at Boston University, Boston, MA, USA following approved standard operating procedures.

### 2.3. RNA Isolation and RT-qPCR

Total RNA was isolated from the lungs of uninfected and SARS-CoV-2-infected hamsters using Trizol reagent and purified by RNeasy mini columns (Qiagen, German Town, MD, USA), as described previously [50]. cDNA synthesis was performed using a High-Capacity cDNA Reverse Transcription Kit with 500 ng of total RNA, as per the standard protocol (ThermoFisher Scientific). Target mRNAs were quantified using Power SYBR Green PCR Master Mix (ThermoFisher Scientific), with the primer sets shown in Supplementary Table S1. The *C_T_* value was normalized to that of GAPDH or β-actin (controls) and represented as a relative value to control using the 2^−ΔΔ^*C_T_* method, as previously described [51,52]. All oligonucleotide primers used in this study were synthesized at Integrated DNA Technologies (IDT, Coralville, IA, USA).

### 2.4. Hamster Infection and Sample Collection

Seventy-five (*n* = 75) male Golden Syrian hamsters (*Mesocricetus auratus*) between 6 and 8 weeks old were purchased (Envigo Corporation, Denver, PA, USA) and housed as two animals/cage. Feed and water were given ad libitum throughout the experiment. The animals were acclimatized for seven days in the BSL3 facilities. Body weight, food, and water intake were monitored twice a day for each animal throughout the experiment. For intranasal infection, SARS-CoV-2 was prepared at 10^2.5^ PFU in 50 µL of sterile PBS. For the uninfected control group, 5 hamsters were intranasally inoculated with 50 µL of sterile 1× PBS, as described previously [47]. At 4, 7, and 16 days post-infection, a group of 5 animals was euthanized and the organs were harvested for histology and immunohistochemistry analysis, as reported previously [47].

### 2.5. Virus Infectivity Assays on Tissues

Tissue homogenates were centrifuged, and the supernatant was filtered through a 0.45 µm filter. The filtrate was diluted in a serum-free DMEM media and 400 µL were used to infect monolayered Vero E6 cells in 6-well plates. Plaque assays were performed as described previously [53]. The plaques were visualized on the third day by staining with 0.2% crystal violet and the numbers of plaque-forming units (PFU) per gram of tissue were determined.

### 2.6. Histopathology Analysis

The tissues were fixed in 10% buffered formalin, made into paraffin blocks, sectioned to 5-micron thickness, and stained with hematoxylin and eosin or Trichrome, as described previously [54]. The identity of the samples was blinded before analysis, and histopathological examination was performed by a board-certified veterinary virologist (S.R.) using the EVOS FL Cell imaging system (ThermoFisher Scientific, Waltham, MA, USA). Histopathology images were organized and labeled using Adobe Photoshop v22.1.1 and Adobe Illustrator v25.1 (Adobe Inc., San Jose, CA, USA). Spleen white pulp cells were quantified manually using ImageJ (NIH, Bethesda, MD, USA). The pulmonary pathology was scored (0–5) based on the degree of mononuclear infiltration, edema, alveolar/bronchiolar hyperplasia, emphysema, vascular lesions, bronchiolar/arteriolar smooth muscle thickening, and foamy macrophages, as reported earlier [47].

### 2.7. Single-Molecule Fluorescence In-Situ Hybridization (smFISH) Probes Synthesis

smFISH probe pools were generated to detect seven different RNA^+^ segments of the SARS-CoV-2 genome (Accession number: NC_045512.2). Each probe pool used (48 probes per pool, each probe 20 nucleotides in length) is listed in Supplementary Table S2. Probes were designed using Stellaris™ probe designer (Biosearch Technologies LGC, Petaluma, CA, USA) and then synthesized and purchased from Biosearch Technologies (the probe sequences are available upon request). The 3′-end of each probe was modified with an amine group and coupled to Texas Red-X (Fisher Scientific, Hampton, NH, USA). Coupled probes were ethanol precipitated and purified on an HPLC column to isolate oligonucleotides linked to the fluorophore, as described previously [55]. The seven sets of probes were diluted to 25 ng/µL, pooled together, and used at 25 ng per hybridization reaction (50 µL). The organization of the SARS-CoV-2 genome and the target region of the smFISH probe are shown in Supplementary Figure S1.

### 2.8. Processing of SARS-CoV-2-Infected THP-1 Cells for Imaging

The SARS-CoV-2 infected THP-1 cells were processed as we reported previously [18]. Briefly, formalin-fixed cells on coverslips were washed once with sterile 1× PBS, and dehydrated by incubating with serial dilutions of ethanol in water at room temperature as follows: 50% ethanol for 5 min; 70% ethanol for 5 min; 100% ethanol for 10 min and stored at 4 °C until ready to use for immunocytostaining.

### 2.9. Processing of Formalin-Fixed Paraffin-Embedded (FFPE) Tissues

Preparation and pre-treatments of tissue specimens for hybridization were carried out as previously described [56,57]. Briefly, FFPE slides were processed sequentially through 100% xylene and 100%, 95%, and 70% ethanol (Fisher Scientific; ethanol solutions were diluted in RNase-free water). Slides were then treated for antigen retrieval with 10 mM citrate buffer (0.173 g citric acid and 2.348 g sodium citrate in 100 mL RNase-free water, pH 6) inside a decloaking chamber (Biocare Medical, Pacheco, CA, USA) at 90 °C for 40 min. Slides were left to cool to room temperature and then fixed with 4% paraformaldehyde.

### 2.10. Immunohistochemistry

Following the deparaffinization of sections, the antigen retrieval was performed using 10 mM citrate buffer at 90 °C for 40 min. The sections were blocked using 1% RNase-free BSA for 1 h and incubated with rabbit anti-hamster ACE2 antibody (R&D Systems, Inc., Minneapolis, MN, USA) or overnight anti-CD31 polyclonal antibody (ThermoFisher Scientific, Waltham, MA, USA) at 4 °C. Alexa Fluor-488 labeled anti-rabbit IgG secondary antibody (ThermoFisher Scientific, Waltham, MA, USA) prepared at 1:2000 dilution in 5% RNase-free BSA was used to detect ACE2 or CD31 expression. The slides were washed with 2× SSC buffer containing 10% formamide in 2× SSC (ThermoFisher Scientific, Waltham, MA, USA), followed by hybridization buffer containing 10% dextran sulfate, 1 mg/mL *E. coli* tRNA, 2 mM ribonucleoside vanadyl complexes (New England Biolabs, Ipswich, MA, USA), 0.02% ribonuclease free BSA (ThermoFisher Scientific, Waltham, MA, USA), 10% formamide, 2× SSC for 30 min. If a multiplex RNA staining was performed, labeled smFISH RNA probes (Biosearch Technologies LGC, Petaluma, CA, USA) were added and incubated overnight in a humidified chamber at 37 °C, as described below.

### 2.11. Hybridization and smFISH Staining

THP-1 cells on coverslips and FFPE tissue sections on glass slides were treated in hybridization wash buffer (10% formamide, 2× SSC) for 20 min. A humidified chamber for hybridization was prepared and pre-warmed at 37 °C, 30 min before hybridization. Each slide was equilibrated with 50 μL of hybridization buffer, which consisted of 10% dextran sulfate (Sigma-Aldrich, St. Louis, MO, USA), 1 mg/mL Escherichia coli transfer RNA (Sigma-Aldrich, St. Louis, MO, USA), 2 mM ribonucleoside vanadyl complexes (New England Biolabs, Ipswich, MA, USA), 0.02% ribonuclease-free BSA (Fisher Scientific), 10% formamide, 2× SSC for 30 min. Another 50 μL of hybridization buffer was prepared per slide along with conjugated probes needed with appropriate concentration (25 ng each of pooled probes) and then placed onto the slides covering the whole tissue section. Slides were placed in the pre-warmed humidified chamber overnight at 37 °C. Following hybridization and washes, slides were then equilibrated with mounting buffer (2× SCC, 0.4% glucose) for 20 min. Equilibrated slides received True Black (Biotium, Fremont, CA, USA) for 1 min and then washed twice with mounting buffer. Slides were mounted in the mounting buffer supplemented with 1 μL of 3.7 mg/mL glucose oxidase and 1 μL of catalase suspension (Sigma-Aldrich, St. Louis, MO, USA) and DAPI (Abcam, Waltham, MA, USA) for each 100 μL preparation. After ensuring all the sections were covered in the mounting medium, a clear coverslip was placed on each slide, sealed with clear nail polish, and then imaged on the same day.

### 2.12. Image Acquisition

Images were acquired using a Zeiss Axiovert 200 M (63× oil immersion objective; numerical aperture 1.4) controlled by Metamorph image acquisition software Version 7.10.5 (Molecular Devices, San Jose, CA, USA). ImageJ/FIJI Version 1.53p was used to analyze the acquired images. Stacks of images of 16 layers with 0.2 µm intervals at 100- to 2000-millisecond exposure times were used in each fluorescence color channel including DAPI. Two representative coverslips per sample/group were selected and 10–20 regions/fields of interest were imaged. Regions of interest were drawn manually around each cell using DIC and DAPI channels, then the average intensity was measured within the area of each cell using RNA-specific fluorescence channels.

### 2.13. Statistical Analysis

Statistical analyses were performed using GraphPad Prism software version 8.0 (GraphPad Software, San Diego, CA, USA). Mean ± standard error (SE) values were plotted as graphs. Unpaired *t*-test with Welch’s correction was used to analyze the data between two groups, and one-way ANOVA with Tukey’s correction was used for multiple-group comparisons. A *p* value less than 0.05 (*p* < 0.05) with 95% confidence intervals, was considered statistically significant for all of the experimental data.

### 2.14. Ethics Statement

All animal procedures were performed in BSL3 facilities according to the ethical policies and procedures approved by the Rutgers University Institutional Animal Care and Use Committee (IACUC Approval no. PROTO202000103), which is consistent with the policies of the American Veterinary Medical Association (AVMA), the United States Center for Disease Control (CDC), and the United States Department of Agriculture (USDA).

## 3. Results

### 3.1. ACE2-Dependent SARS-CoV-2 Infection of Cultured Human Macrophages Modulates the Expression of Key Homeostasis Pathways

Macrophages and myeloid lineage cells are not the primary targets of SARS-CoV-2 infection. However, ACE2 expression on alveolar and blood-derived macrophages is well established in patients during SARS-CoV-2 infection [58,59]. As shown in a recently established system comprising primary and cultured macrophages, expression of ACE2 supports productive viral infection and proinflammatory cytokine responses that may shape the outcome of infection [18]. The interactions of ACE2 with other key molecular pathways may support the role of macrophages in homeostasis but currently remain largely unknown. Therefore, we used human macrophages expressing ACE2 to investigate the effects of proinflammatory responses induced by SARS-CoV-2 on ACE2, CD147, and GRP78 molecular pathways, which are crucial to maintaining homeostasis in the lung.

**Figure 1:**
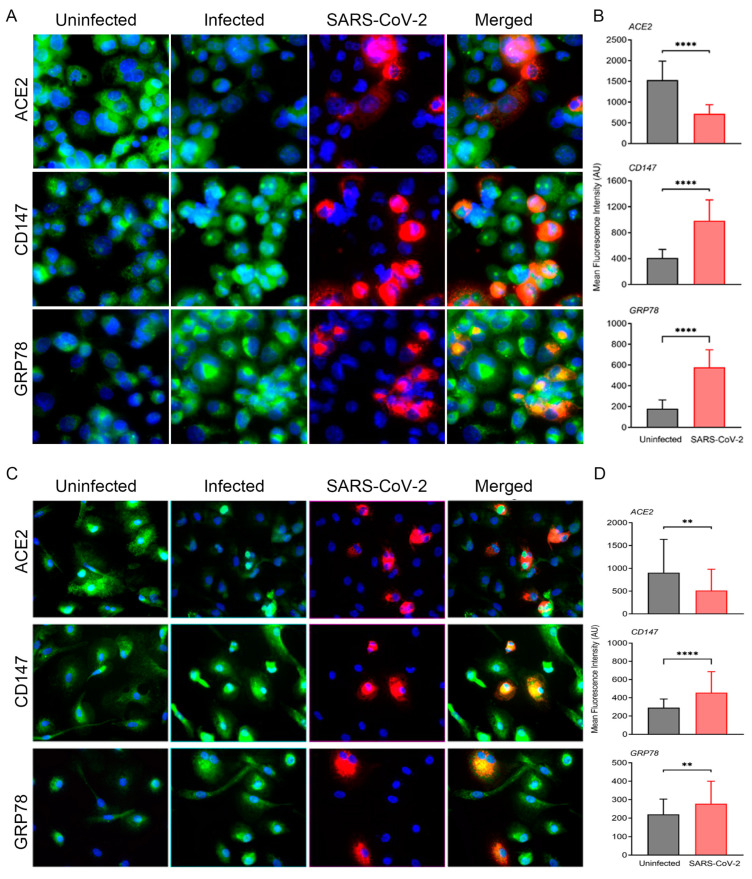
SARS-CoV-2 infection modulates ACE2, CD147 and GRP78 pathways in THP-1/ACE2 and in human MDM/ACE2 macrophages. (**A**) Representative images of PMA-differentiated THP-1 macrophages transduced with ACE2 with or without SARS-CoV-2 infection (at 5 MOI). The images were taken at 24 hpi with 63× magnification and the scale bar represents 20 μm. Virus (red)- and host cell marker (green)-specific smFISH and nuclear DAPI-staining (blue) are shown. (**B**) Quantitative measurements of host marker expression from at least 50 cells per field (n = 3 fields per sample with at least 2 samples per condition), normalized against background fluorescence. (**C**) Representative images of human monocyte-derived macrophages (MDM) transduced with ACE2 and with or without SARS-CoV-2 infection (at 5 MOI). The images were taken at 24 hpi with 63× magnification and the scale bar represents 20 μm. Virus (red)- and host cell marker (green)-specific smFISH and nuclear DAPI-staining (blue) are shown. (**D**) Quantitative measurements of host marker expression from at least 50 cells per field (n = 3 fields per sample with at least 2 samples per condition), normalized against background fluorescence. Values plotted are mean ± SD (n = 3–4 coverslips per group). Only statistically significant differences were shown in the figure. ** *p* < 0.01 and **** *p* < 0.0001 by unpaired *t*-test.

ACE2-transduced, PMA-differentiated THP-1 macrophages and MDMs were infected with SARS-CoV-2 and analyzed by smFISH using color-distinguishable probes for cellular markers and viral RNA (Figure 1A–D). At 24 h post-infection (hpi), expression of ACE2-specific RNA was downregulated in infected cells compared to the mock-infected cells, whereas RNA levels of CD147 and GRP78 molecules were upregulated in both MDMs and THP1/PMA macrophages. The mechanisms underlying these changes in RNA expression remain undefined but could differ significantly among these key molecules. In general, but also as shown for ACE2 tested in more than 150 human cell types corresponding to all major tissues, there is a good correlation between the levels of mRNA transcripts and protein [60]. Expression of ACE2 protein may be modulated by increased shedding under a variety of pathophysiological conditions including SARS-CoV-2 infection [26]. Modulation of protein expression may transduce signals that alter transcriptional controls resulting in increased or decreased RNA expression. The modulation of RNA levels of ACE2, CD147, and GRP78 was observed in both infected and non-infected bystander cells, suggesting that virus-associated secreted factors might play a role.

### 3.2. SARS-CoV-2 Infected Hamsters Show Pathophysiology and Altered Homeostasis Resembling That of COVID-19 Patients Undergoing Proinflammatory and Prothrombotic Shifts

To further evaluate the effects of SARS-CoV-2 infection and modulation of homeostasis in the lung, we employed a hamster model of infection [47]. Following intranasal infection, all infected hamsters survived until experimental euthanasia. Infected hamsters showed a reduction in body weight from the day of infection up to 6 days post-infection (dpi) (Supplementary Figure S2A). The animals then gradually gained weight from 8 to 16 dpi, suggesting that the animals experienced inappetence or anorexia in the early stages of infection.

The viral burden (PFU) in the lungs of infected hamsters was also measured following SARS-CoV-2 infection at multiple time points. The viral load began to peak at 2 dpi reaching the highest levels at 4 dpi, but then statistically significantly decreased at 7 dpi and disappeared by 12–16 dpi (Supplementary Figure S2B). This was confirmed by measuring SARS-CoV-2 transcripts using RT-PCR in the lungs of infected hamsters from 2 to 16 dpi with the infection peak detected at 2–4 dpi (Supplementary Figure S3).

Next, we determined the severity of pulmonary pathology and thrombosis in the lung induced by SARS-CoV-2 infection. Representative H&E histologic images of lung sections from SARS-CoV-2 infected hamsters at 4 dpi showed key pathological features related to COVID-19 in humans (Supplementary Figure S2). Thus, SARS-CoV-2 infected hamster lungs exhibited loss of endothelial cells in the vasculature, adherence of red blood cells (RBCs) on the wall of the blood vessel, extravasation of platelets in the pulmonary tissues, and red-thrombus tightly adhered to the walls of pulmonary arterioles, occluding the lumen (Supplementary Figure S2). Loss of endothelial cells in the vasculature, prothrombotic changes in RBCs, as well as platelet migrations are likely to underscore the formation of thrombi leading to thrombosis.

### 3.3. Expression of ACE2 in Hamster Lungs following SARS-CoV-2 Infection Is Downregulated in the Cells Harboring the Virus

**Figure 2:**
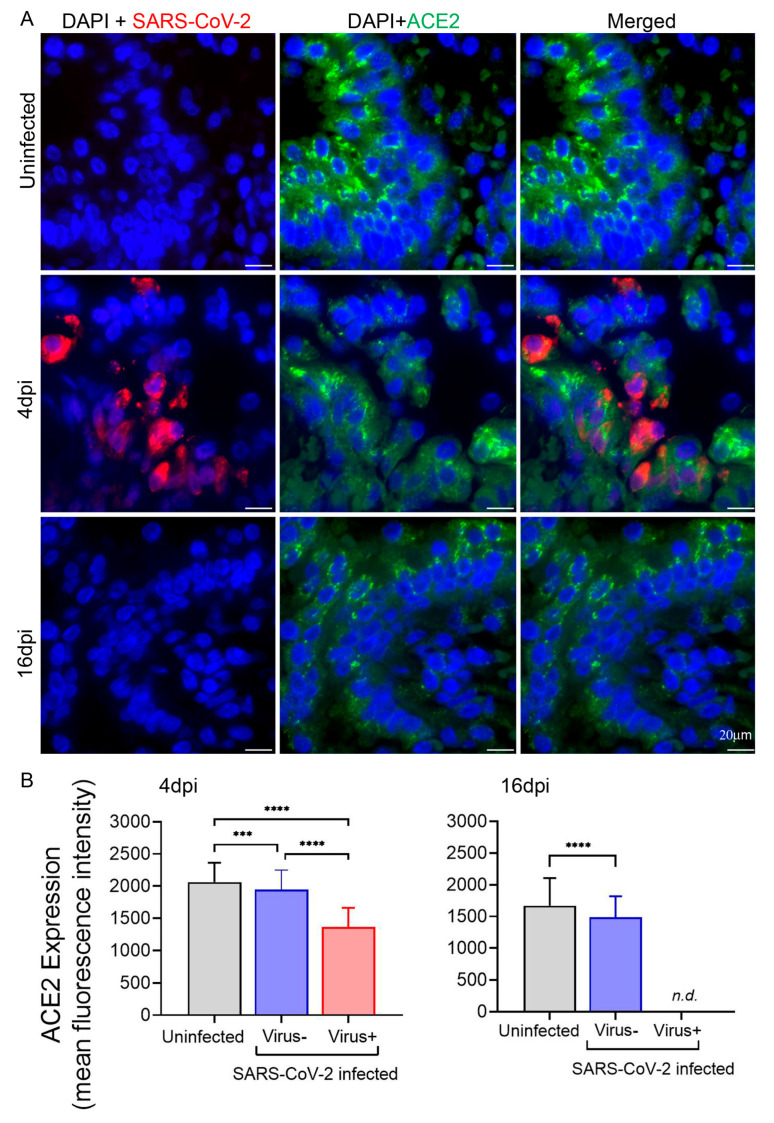
ACE2 expression is differentially modulated in hamster lungs during pulmonary SARS-CoV-2 infection. (**A**) Representative images of hamster lung sections hybridized with probes targeting SARS-CoV-2 (red) and ACE2 (green) mRNA and nuclear DAPI-staining (blue), respectively, at 4 and 16 dpi. Uninfected lung sections are included as control. Images were acquired at 63× magnification, and the scale bars represent 20 μm. Each image analyzed was at the maximum intensity of merged z-stacks with 300–500 cells analyzed for each group. (**B**) Expression of ACE2 in the hamster lungs with or without SARS-CoV-2 infection at 4 and 16 dpi as measured by mean fluorescence intensity; n.d.—virus not detected. Values plotted are mean ± SD (n = 3–4 animals per group). Only statistically significant differences were shown in the figure. **** *p* < 0.0001 and *** *p* < 0.001 by unpaired *t*-test.

To explore the modulation of ACE2 expression and spatial distribution of ACE2 along with SARS-CoV-2 transcripts at the single cell level, smFISH analysis was performed with infected hamster lung samples stained simultaneously with two probe sets. There was a significant reduction in the expression of ACE2 mRNA in infected hamster lungs at 4 dpi (Figure 2A). The downregulation of ACE2 expression was reversed, but not yet fully restored, by 16 dpi. Interestingly, the determination of ACE2 expression levels in the cells harboring viral RNA versus those with undetectable virus at the peak of infection revealed a more dramatic downregulation of ACE2 in infected cells, whereas bystander cells showed an ameliorated decrease in ACE2 expression (Figure 2B, left panel).

**Figure 3:**
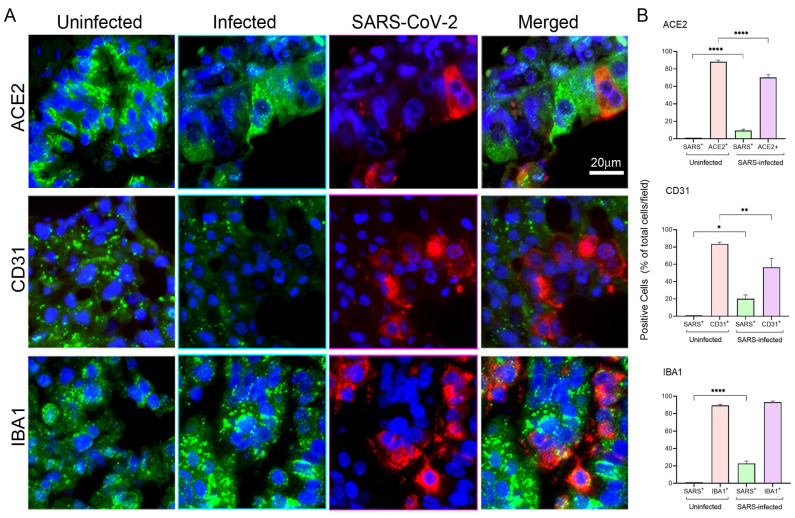
Differential expression patterns of ACE2, CD31, and IBA1 markers underscores cell type-specific susceptibility of SARS-CoV-2 infection in hamster lungs. (**A**) Representative images of hamster lung sections showing positive signals of SARS-CoV-2 transcripts (red) and antibody-stained ACE2, CD31, or a macrophage activation marker, IBA1 (green), and nuclear DAPI-staining (blue), respectively. Images were acquired at 63× magnification, and the scale bars represent 20 μm. Each image analyzed was at the maximum intensity of merged z-stacks with 370–550 cells per field and 10 fields of each section were analyzed. (**B**) Percentage of cells positive for SARS-CoV-2 and ACE2, CD31, or IBA1 among the total cells in uninfected and infected hamster lung sections. Values plotted are mean ± SD (n = 3–4 animals per group). Only statistically significant differences were shown in the figure. * *p* < 0.05, ** *p* < 0.01 and **** *p* < 0.0001 by unpaired *t*-test.

Further investigation of modulations of ACE2 expression included the evaluation of protein levels in hamster lungs at 4 dpi by anti-hamster ACE2 antibody staining (Figure 3A, top panels). In these experiments utilizing Ab-staining, we quantified the number of cells expressing markers specific to cell types common in the lung. Consistent with the expression of RNA observed by smFISH analysis, diminished ACE2 protein expression in infected lungs was accompanied by a statistically significant reduction of ACE2+ cells (Figure 3B, top panel). The downregulated ACE2 expression was likely observed in epithelial cells since at least some of these cells co-expressed viral RNA and because endothelial and macrophage cells also present in the lung seldom had SARS-CoV-2 RNA (see below).

To further determine whether the endothelial and macrophage cells in the hamster lung could harbor SARS-CoV-2, multiplexed staining with cell type-specific Abs, anti-CD31 and anti-IBA1, to identify endothelial cells and macrophages, respectively, and SARS-CoV-2-specific smFISH probes were carried out at 4 dpi (Figure 3A, middle and lower panels). CD31 is a cellular marker of vascular differentiation, and is highly expressed on endothelial cells, but to a lesser degree on platelets, monocytes, and granulocytes. The staining produced two mutually excluding, differentially colored cell populations: either green (expressing cellular markers) or red (virus-bearing). Accordingly, co-expression of markers and virus could be detected by observing pink rather than red-colored cells. CD31^+^ cells were seen in lung sections where the cells with the most highly detectable SARS-CoV-2 RNA signal were observed. As with ACE2+ cells, infection reduced the presence of detectable CD31+ cells, although this was less statistically significant (Figure 3B, middle panel). Downregulation of CD31 expression in the endothelium in response to proinflammatory cytokines has been documented previously [61,62]. In contrast, although IBA1 staining in infected versus uninfected lungs revealed no significant changes in the number of resident macrophages (Figure 3B, lower panel), increased intensity of IBA1 staining in infected lung tissues was observed, which might indicate a more activated phenotype of these cells (Figure 3A, lower panel). Additionally, image analysis revealed that CD31^+^ cells in the lungs did not co-express SARS-CoV-2 while only a few IBA1+ cells appeared to have viral RNA expression (Figure 3A, middle and lower panels).

In summary, these tissue immunostaining analyses revealed that, similar to in vitro findings, there was a marked downregulation of ACE2 expression in viral RNA+ cells, Furthermore, there was a virtual absence of viral RNA expression in ACE2-deficient CD31+ cells or IBA1+ macrophages.

### 3.4. SARS-CoV-2 Infection Upregulates Expression of CD147 and GRP78 in Hamster Lung Tissues

**Figure 4:**
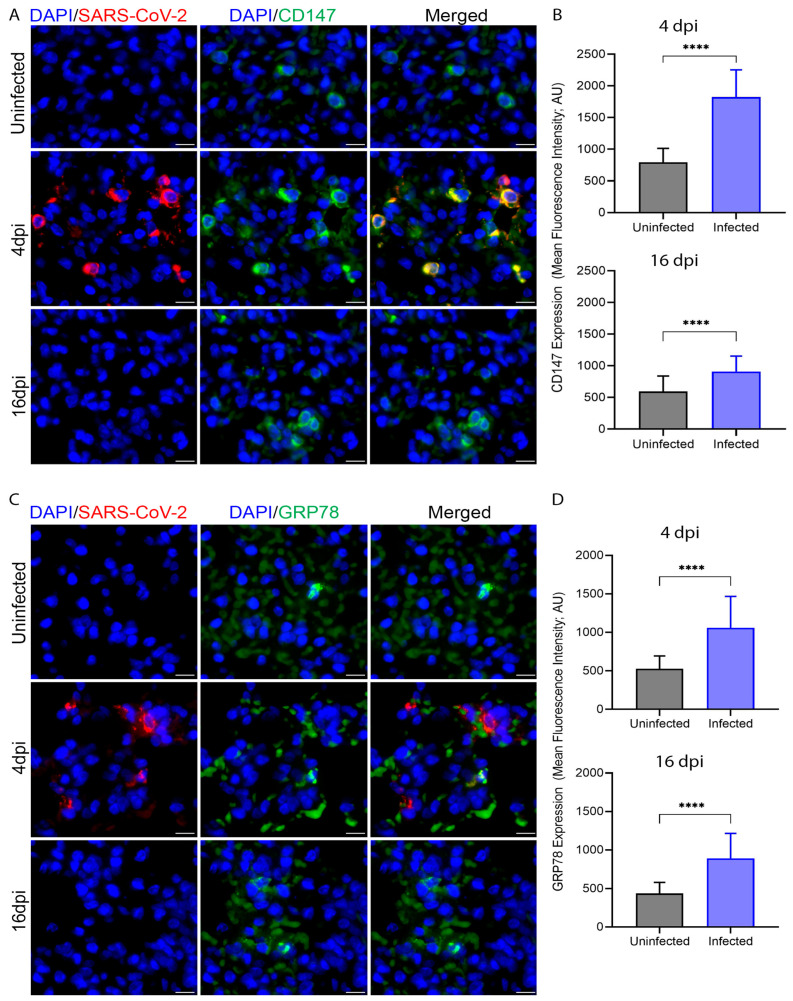
Expression of CD147 and GRP78 is upregulated in hamster lungs during SARS-CoV-2 infection. (**A**) Representative fields of hamster lung sections hybridized with probes targeting SARS-CoV-2 and CD147 mRNA, respectively. Each image is a maximum intensity merged z-stacks. Microscopy at 63× with scale bars representing 20 μm. (**B**) Upregulation of CD147 expression in the lung of infected hamster at 4 and 16 dpi. (**C**) Representative fields of hamster lung sections hybridized with probes targeting SARS-CoV-2 and GRP78 RNA, respectively. Each image is a maximum intensity merged z-stacks. Microscopy at 63× with scale bars representing 20 μm. (**D**). Upregulation of GRP78 expression in the lung of infected hamster at 4 and 16 dpi. Between 300–500 cells were analyzed for each group. Values plotted are mean ± SD (n = 3–4 animals per group). Only statistically significant differences were shown in the figure. **** *p* < 0.0001 by unpaired *t*-test.

CD147 and GRP78 molecules are crucial in maintaining lung immune functions and vascular homeostasis [36,37,40,42,43]. Also, CD147 and GRP78 may function as alternate receptors for SARS-CoV-2 [63,64]. The levels of expression of both molecules in the infected hamster lungs were determined by smFISH analysis. There was a dramatic upregulation in the expression of CD147 and GRP78 RNAs upon virus infection, peaking at 4 dpi (Figure 4). Upregulation of CD147 was mostly observed in virus-harboring cells, although a similar effect on bystander cells could not be ruled out, while co-expression of GRP78 and viral RNA was less prominent. This conclusion is supported by the observation that following the resolution of infection at 16 dpi, expression of these molecules remained significantly higher than baseline levels (Figure 4). The bystander effect of SARS-CoV-2 infection was also observed in virus-infected human macrophages, thus indirectly confirming that altered expression is associated with infection and is not an attribute of the lentiviral expression system (Figure 1). The spatial compartmental distribution of these host cell transcripts relative to viral transcripts may be different and may affect the color intensities of corresponding markers on merged images. Furthermore, the levels of induction, which are different for upregulated CD147 and GRP78 in infected lungs may indicate different mechanisms modulating the expression of these molecules. Upregulation of CD147 during inflammation mainly occurs through transcriptional synthesis and generation of membrane-bound or soluble proteins while upregulation of GRP78 results from the release of bound proteins from ER stores and a concomitant increase of RNA expression [38,40,41]. The persistently increased levels of CD147 and GRP78 expression may suggest an elevated inflammatory state of the alveolar cells and a profound effect of SARS-CoV-2 infection on homeostasis.

### 3.5. SARS-CoV-2 Infection Upregulates the Expression of Coagulation Cascade Markers That Reveal the Prothrombotic Shift in Hamster Lungs

COVID-19 is frequently associated with microvascular thrombosis, especially in the lung, or macrovascular thrombosis (thromboembolism), which contributes to the severity of the disease [65]. We determined the prothrombotic events in hamster lungs caused by SARS-CoV-2 infection by evaluating RNA expression of coagulation cascade markers TF, plasminogen activator (PLAT), plasminogen activator inhibitor-1 (PAI-1), thrombomodulin (THBD), and vWF using smFISH, RT-PCR, and RNAseq methods.

**Figure 5:**
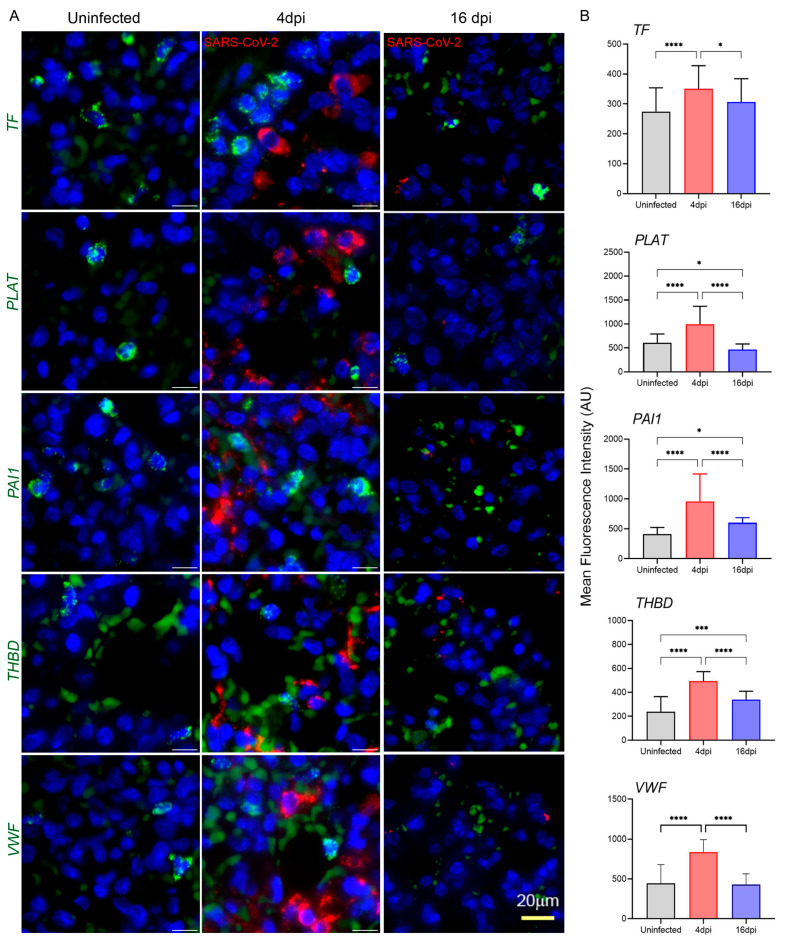
Spatial analysis of prothrombotic and coagulation cascade marker expression in hamster lungs during SARS-CoV-2 infection. (**A**) Representative images of hamster lung sections hybridized with probes targeting TF, PLAT, PAI1, THBD and VWF mRNA respectively. Each image is a maximum intensity merged z-stacks. Microscopy at 63× with scale bars representing 20 μm. (**B**) Quantitative analysis of the level of expression of selected genes in prothrombotic and coagulation cascade (TF, PLAT, PAI1, THBD, and VWF). For each plot, at least 50 cells were analyzed and normalized against background fluorescence. Significant differences between the groups were determined by one-way ANOVA with Tukey’s multiple comparison tests showing the mean fluorescence intensity of each marker ± SD in each group (3–4 animals per group). Only statistically significant differences were shown in the figure. * *p* < 0.1, *** *p* < 0.001, and **** *p* < 0.0001.

The smFISH analysis revealed that all of the coagulation cascade markers tested in this study showed significantly upregulated expression at 4 dpi as seen in representative images and by quantification (Figure 5A,B).

**Figure 6:**
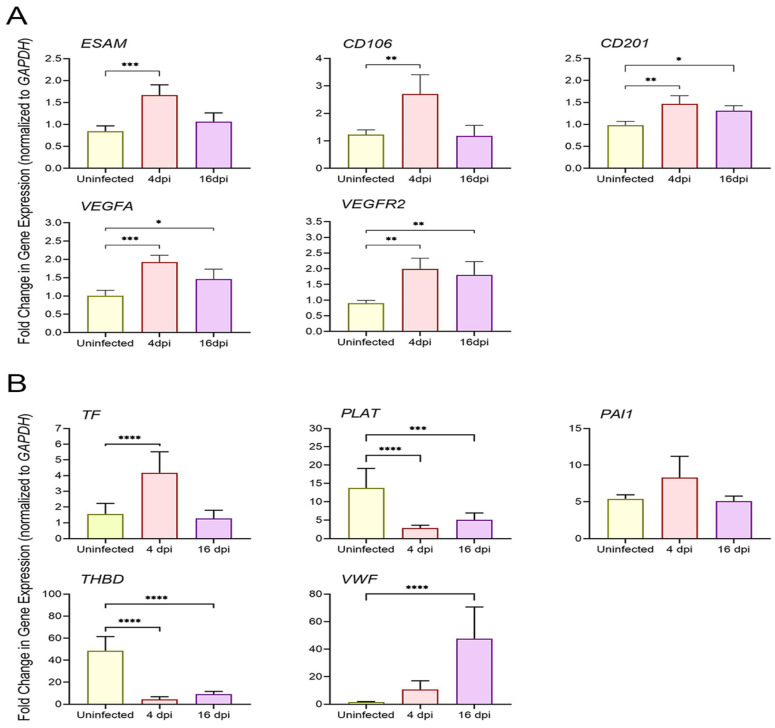
Expression of prothrombotic shift, coagulation cascade, and endothelial markers in hamster lungs during SARS-CoV-2 infection by RT-PCR. (**A**) Fold change in the expression of marker genes TF, PLAT, PAI-1, THBD, and vWF in uninfected, 4 or 16 dpi hamster lungs. (**B**) Expression of endothelial marker genes ESAM, CD106, CD201, VEGFA, and VEGFR2 in uninfected, 4 or 16 dpi hamster lungs. Significant differences between the groups were determined by one-way ANOVA with Tukey’s multiple comparison tests showing fold change in gene expression ± SD for each group in 2 independent experiments (3–4 animals per group). Only statistically significant differences were shown in the figure. * *p* < 0.1, ** *p* < 0.01, *** *p* < 0.001, and **** *p* < 0.0001.

No clear evidence of co-expression of coagulation cascade markers and SARS-CoV-2 RNAs in images was found (Figure 5), which may indicate that the infection-induced enhancement of cellular markers occurs in neighboring cells regardless of their infection status. The upregulated RNA expression of coagulation cascade markers was reversed at 16 dpi and decreased to near baseline for PLAT, PAI-1, and vWF, or remained slightly elevated for TF and THBD (Figure 5A,B). Transient upregulation of anti-coagulant PLAT and THBD was unexpected (see below). These results correlated with the high level of viral RNA expression at 4 dpi, which was completely reduced by 16 dpi, further underscoring the roles of the virus-induced inflammation state and cellular stress as a possible link to the increased levels of CD147 and GRP78. However, the RT-PCR results obtained with total lung RNA samples were not fully consistent with the observations of smFISH analysis (Figure 6A,B). TF upregulation was observed at 4 dpi, followed by a return to baseline levels at 16 dpi (Figure 6A). In contrast to SARS-CoV-2-induced transient upregulation of PLAT and THBD expression at 4 dpi (smFISH data), expression of anti-coagulant PLAT and THBD was suppressed in lung tissues at all tested time points post-infection, which, together with elevated TF, could be expected at the prothrombotic stage of infection. PAI-1 and vWF responded to infection differently, showing a trend of slight upregulation in PAI-1 at 4 dpi and delayed upregulation of vWF at 16 dpi (Figure 6B).

Overall, the data showed that, among the tested genes, TF was the only reliable marker of thrombosis. The upregulation of TF at 4 dpi and return to basal levels at 16 dpi was confirmed by both spatial imaging and RT-PCR. This conclusion underscores the differences in the sensitivity and specificity thresholds of methods employed to study transcript expression.

### 3.6. SARS-CoV-2 Upregulates Markers of Endothelial Dysfunction in Infected Hamster Lungs

SARS-CoV-2 is known to cause endothelial dysfunction leading to impaired vascular activity and multiorgan injury [21]. Using total RNA collected from the lungs of SARS-CoV-2-infected hamsters at 4 and 16 dpi points, we used RT-PCR to measure the expression levels of known specific markers indicating the health and performance (inactive or procoagulant) states of the endothelium [66]. The targeted markers, ESAM, CD106, CD201, VEGF-A, and VEGFR-2, whose elevated expression is associated with inflammation and endothelial dysfunction were indeed upregulated at 4 dpi (Figure 6A). Resolution of infection at 16 dpi resulted in downregulation of their expression, except for CD201, VEGF-A, and VEGFR-2, which remained elevated above baseline levels seen in uninfected lungs (Figure 6A). These findings may indicate a pathophysiological state in the hamster lung following SARS-CoV-2 infection, where increased endothelial inflammation leads to endothelial leakage and inflammatory cell infiltration.

## 4. Discussion

Although primarily targeting respiratory organs, SARS-CoV-2 can have significant detrimental effects on the cardiovascular system [63]. Severe cases of infection frequently result in respiratory distress, potentially because the virus can enter the circulation system through lung alveoli. The pathophysiology of COVID-19 set in motion directly (by virus infection) and indirectly (by cytokine storm) affects homeostasis and endothelial performance [21]. Clinical manifestations such as vasculitis, have been detected in multiple vascular organs including lungs, heart, and kidneys, with thromboembolism being observed in several COVID-19 patients, suggesting that infection activates endothelial cells via immune-mediated inflammatory responses, perturbs the vasculature and endothelial homeostasis, thus leading to vascular dysfunction [21,67,68]. Therefore, COVID-19 is considered to be a vascular and endothelial disease.

There is a growing body of evidence that ACE2, CD147, and GRP78 pathways are involved in the regulation of lung homeostasis. The underlying molecular mechanisms perturbed by SARS-CoV-2 infection may cause the vascular dysfunction seen during COVID-19. However, mechanistic links connecting these regulatory pathways with the coagulation cascade and endothelial performance pathways and the molecular events causing endothelial dysfunction remain unknown. This study demonstrates that SARS-CoV-2 modulated the expression of key molecules, namely, the downregulation of ACE2 and upregulation of CD147 and GRP78, which was observed upon infection of human cell cultures and when using a hamster model of pulmonary infection.

Activated monocytes and macrophages with traces of viral antigens were found in post-mortem samples and dysfunctional monocytes and macrophages have been associated with an increased proinflammatory state in COVID-19 patients [69,70,71]. The human in vitro system used in this study utilized cultured macrophages designed to express ACE2 levels sufficient for productive SARS-CoV-2 infection [18]. This system provided initial observations of significant changes in the expression of ACE2, CD147, and GRP78 molecules induced by infection (Figure 1). With the hamster model of SARS-CoV-2 infection, expression of ACE2, CD147, and GRP78 molecules and cellular markers relevant to the activation/prothrombotic state and endothelial performance was determined in lung tissues by RNA and protein detection methods. Histological analyses of lung tissues from infected animals showed a mild-to-moderate COVID-19 pathophysiology with the formation of thrombi on pulmonary vessel walls and loss of endothelial cells in the vasculature at the peak of infection (Supplementary Figures S2 and S3). RNA expression patterns of ACE2, CD147, and GRP78 in infected lung tissues confirmed the downregulation for ACE2 and upregulation of CD147 and GRP78 (Figure 2 and Figure 4), similar to the outcome seen with human macrophages. Dampened ACE2 expression in this system is consistent with previous flow cytometry-based findings of significantly reduced membrane-bound ACE2 in monocyte/dendritic cells of COVID-19 patients [72]. Based on staining patterns with cell type-specific Abs (Figure 3), we noticed that modulations of ACE2, CD147, and GRP78 determined by smFISH are likely to occur in lung epithelial cells. These observations may suggest yet undefined mechanisms that facilitate a prothrombotic shift in vivo during infection. Thus, a population of infected macrophages interacting with endothelial cells is likely to promote thrombosis.

Since histopathology revealed signs of thrombosis and endothelial stress in pulmonary vessels of infected animals, we next investigated whether SARS-CoV-2 infection perturbed homeostasis and the resulting prothrombotic shift might reveal characteristic markers detectable by smFISH. Indeed, the salient finding was a significant upregulation in the expression of coagulation cascade markers such as TF and vWF upon SARS-CoV-2 exposure (Figure 5). This uptick in expression correlated well with the infection phase, i.e., maximizing at the peak and getting back to base levels after the resolution of infection. A similar dependence on the infection phase was observed with the ACE2, CD147, and GRP78 expression patterns. Of note, the increased expression of some of these coagulation cascade markers, such as TF and PAI-1, was recently observed using various experimental systems probing the effect of SARS-CoV-2. This includes human endothelial cells infected with viral spike 1 protein and SARS-CoV-2 spike pseudovirions (SCV-2-5) or when the cells were treated with recombinant SARS-CoV-2 S protein [73,74,75]. 

This study had certain limitations due to the variability of the applied methodology. Thus, the smFISH data (Figure 5B) was not consistent with the RT-PCR results (Figure 6B), which could be due to the inherent bias in tissue sampling associated with these detection methods. For smFISH, the lung segments with detectable virus were analyzed, while total RNA from the whole minced lung was used in RT-PCR. Furthermore, while smFISH is focused on transcripts expressed in individual cells, RT-PCR determinations were made irrespective of the lung cellular composition.

However, total lung RT-PCR successfully identified markers associated with endothelial stress and dysfunction. We found that the expression of ESAM, CD106, CD201, VEGF-A, and VEGFR-2 was significantly upregulated (Figure 6A) in correlation with the course of infection peak and modulations of ACE2, CD147, and GRP78 expression levels. The roles of the coagulation cascade pathways in homeostasis and the main steps leading to thrombosis have been elucidated [13]. However, the molecular mechanisms linking the prothrombotic shift and endothelial dysfunction in the setting of SARS-CoV-2 infection have yet to be characterized. It has been shown, however, that treatment of human pulmonary microvascular endothelial cells with SARS-CoV-2 S1 subunit or S protein decreased the endothelial barrier function and caused vascular leakage involving integrins and transforming growth factor-beta (TGF-β) signaling [76,77]. Excessive inflammation in the lung induced by SARS-CoV-2 infection is well established [5,6]. Among the members of upregulated markers on endothelial cells, ESAM and CD106 are known to participate in cell-to-cell contact and mediate the adhesion of immune cells to vascular endothelium during inflammation [9,66]. Another endothelial-specific marker upregulated by infection, CD201, binds to Mac-1 (CD11b/CD18) on monocytes, mediating interactions with monocytes. It may also regulate thrombosis by enhancing the activation of protein C interacting with thrombi-thrombomodulin molecular complexes [66,78]. The VEGF-A/VEGFR-2 complexes, which were also upregulated by infection, mediate angiogenic functions in healthy endothelium. However, increased expression of VEGFR-2 or activation of the VEGFR-2-dependent signaling pathway leads to rupture of cell-cell interactions via the activation of VE-cadherin and causes increased endothelial permeability in the microvascular bed [79,80].

As demonstrated here, SARS-CoV-2 infection has a profound effect on the three multifunctional homeostatic ACE2, CD147, and GRP78 pathways. These pathways are ubiquitously expressed, including in the airway epithelial cells of the lung [81]. While the molecular interactions with signaling pathways underlying homeostasis and endothelial health are still unknown, this study identified important markers of prothrombotic shift and endothelial performance. The prothrombotic shift in infected lungs was revealed by increased expression of the coagulation cascade mediators TF, PLAT, PAI-1, THBD, and vWF associated with endothelial dysfunction as shown through the upregulated expression of ESAM, CD106, CD201, VEGF-A, and VEGFR-2. Collectively, these markers correlated with the modulation of ACE2, CD147, and GRP78 expression patterns and the infection states in human macrophage and hamster pulmonary infection models.

ACE2 may have the main role in initiating the prothrombotic shift and loss of endothelial protection among these three key pathways. The outcome of the interaction between ACE2 and SARS-CoV-2 virus, and the regulation of ACE2 expression are still debated [27,30,82,83,84,85]. Although the roles of positive feedback loops should also be considered [86], this aspect is complex due to the intricate interplay of the multiple factors involved, such as activities of membrane vs. soluble ACE2 forms, intracellular ACE2 degradation pathways, tissue specificity, and infection sites analyzed. We did not investigate these mechanistic aspects in this article. Another limitation is that the upregulation of coagulation cascade markers was accessed on lung tissues without specific identification of cell types involved. While these markers are expressed on several immune and endothelial cells, lack of commercially available verified antibodies to use in hamster tissues precluded a more detailed analysis.

Nonetheless, downregulation of membrane-bound ACE2 as seen in this study and upregulation of soluble ACE2 as reported by others are not incompatible findings. Transcriptomic analysis and single cell RNAseq profiling of ACE2, combined with imaging-based proteomics of human body tissues showed the highest expression in the intestinal tract followed by kidney, testis, gallbladder, and heart and low expression in specific cell types of the lung and respiratory epithelia [60,87]. Considering the fact that expression of circulating, enzymatically active ACE2 is generated mostly by the proteolytic cleavage of the membrane-bound form [30,31,34], it is reasonable to speculate that the bulk of increased soluble ACE2 in the blood and other body fluids of COVID-19 patients is not necessarily released from the primary sites of infection, such as upper respiratory tract and lungs. It may be shed from other non-virus-harboring tissues that are still indirectly affected by the infection. Whether the signaling through ACE2 in infected vs. non-virus-harboring cells is different remains to be established. Modulation of ACE2 expression by SARS-CoV-2 at the sites of infection, e.g., lung, may alter its activity and related pathways. In our hamster model of mild-to-moderate COVID-19, we analyzed the lung tissues and found intracellular ACE2 expression downregulated at mRNA and protein levels (Figure 2 and Figure 3A). In fact, intratracheal administration to hamsters of a pseudovirus expressing S protein alone was sufficient to downregulate ACE2 in the lung with consequently impaired mitochondrial function and damaged vascular endothelium [88]. In contrast to these data, the post-mortem immunochemistry of lung tissues from patients with severe COVID-19 showed increased levels of intracellular ACE2 proteins in comparison to non-COVID-19 patients [85]. In a healthy state, ACE2 exerts anti-thrombotic effects through multiple mechanisms involved in the RAS pathway [23,89,90]. At the site of infection, downregulation of ACE2 expression may increase vascular permeability, leading to TF expression in subendothelial cells as well as in leucocytes and platelets, which can trigger disseminated coagulation and thrombosis in infected lungs. Upregulation of CD147 and GRP78 may be consequential to the initial signaling through ACE2 and exacerbate the proinflammation and cellular stress ultimately resulting in endothelial dysfunction. In summary, the data suggest that the signaling via these pathways may be modulated by inflammation associated with infection and play crucial roles in thrombotic control and maintenance of endothelial integrity. Further research is necessary to unravel the mechanistic links connecting the homeostatic pathways identified by this study.

## Data Availability

All the experimental data are presented as figures and tables in this manuscript. Raw data of the figures and tables can be obtained from the lead contact upon formal request. This article does not report any original code.

## Supplementary Materials

The following supporting information can be downloaded at: https://www.mdpi.com/article/10.3390/cells13050432/s1, Table S1. List of the hamster gene-specific primer sequences used for RT-PCR studies; Table S2. List of the genes and corresponding probe sequences used for smFISH studies; Figure S1. Organization and expression of SARS-CoV-2 complete genome and FISH probe diagram targeting its genes; Figure S2. Clinical pathology of SARS-CoV-2 infected hamsters; Figure S3. Number of total viral RNA copies in the lung of SARS-CoV-2 infected hamsters.
