# Supplementary material for: Modulations of Homeostatic ACE2, CD147, GRP78 Pathways Correlate with Vascular and Endothelial Performance Markers during Pulmonary SARS-CoV-2 Infection
Source: Cells. 2024 Feb 29;13(5):432. doi: 10.3390/cells13050432 (PMC10930588; doi:10.3390/cells13050432)
Supplement: Supplementary file 1 [file cells-13-00432-s001.zip › cells-2883407-supplementary.pdf]

Supplementary Materitals

Modulations of Homeostatic ACE2, CD147, GRP78 Pathways Correlate with Vascular and Endothelial Performance Markers during Pulmonary SARS-CoV-2 Infection

Annuurun Nisa[1], Ranjeet Kumar[1], Santhamani Ramasamy[1, ‡], Afsal Kolloli[1], Judith Olejnik[2,3]
Sallieu Jalloh[2], Suryaram Gummuluru[2], Selvakumar Subbian[1,*], Yuri Bushkin[1,*]

Supplementary Table S1. List of the hamster gene-specific primer sequences used for RT-PCR studies.

Gene name		Gene sequence (5′ – 3′)
ACE2	Forward	GGGACGCTACCATTCACTTAC
	Reverse	GCTTCATCTCCCACCACTTT
CD147	Forward	GACTATGCCTGCATCTTCCTT
	Reverse	ACTGGAATGCTCTGACTTCTTC
GRP78	Forward	TCTTGTCGGTGGGTCTACT
	Reverse	ACAGCCTCATCTGGGTTTATG
TF	Forward	CCTATCTGTCCCACGGAATAAC
	Reverse	GGATTACAGGCTTTCCGAGTT
PLAT	Forward	GGAAGAAGTGAGGACTGCTATG
	Reverse	TGCTGTGTAACTCTTGCCTATC
THBD	Forward	GGATCCGTGCTTCGGAAATA
	Reverse	CCTCGGGTTTGGGTCTAAAG
vWF	Forward	GATGTACGGTCTCTGTGGAATC
	Reverse	GCACAGTCCATTCCTGGATAA
PAI-1	Forward	CGTGGAACCAGAACGAGATTAG
	Reverse	GTCCGGAAGAGCTTGAAGAAG
ESAM	Forward	GCCACCCTTGTCAATAGCCT
	Reverse	GCGAGGGAAGCAAAGGTACT
PROCR/CD201	Forward	ACCTGTGTGCAGTTTGTGGA
	Reverse	GAAGTGTAAGAGCGGCCTGT
VCAM-1/CD106	Forward	TCCATTTCGTCACCACAGCA
	Reverse	AGCTTGAGAGGCTGCAAACA
VEGF-A	Forward	TGCATTGGACCCTGGCTTTA
	Reverse	CTGCTCTCCTTCTGTCGTGG
VEGFR-2/CD309	Forward	CAGTGGGATGGTCCTTGCAT
	Reverse	GTACCCAGACTGATAGCCGC
GAPDH	Forward	AGAACGGGAAGCTTGTCATC
	Reverse	GCCAGTAGACTCCACAACATAC
β-actin	Forward	CCCAGAGCAAGAGAGGTATTC
	Reverse	GGTGCCAGATCTTCTCCATATC

Supplementary Table S2. List of the genes and corresponding probe sequences used for smFISH studies.

**SARS-CoV-2 #1**	**SARS-CoV-2 #2**	**SARS-CoV-2 #3**
tgagttggacgtgtgttttc	atagccttgtatcctatgat	agctgcatatgatggaaggg
gcgaacctgtaaaacaggca	atgctatgtcacgagtgaca	gccacattcaaagacttctt
ccataacatgaccatgaggt	atcaatgggcaagctttgtc	ctgcatcacggtcaaattca
tactgaatgccttcgagttc	cttctcttgttatgactgca	tcttttccaacttacgttgc
aatgcactcaagagggtagc	tcaccattagttgtgcgtaa	ttgggtcatagcttgatcag
ccagttgttcggacaaagtg	cagatgttaccaactgcact	tcagatctagcctgtttata
tttcagaacgttccgtgtac	gtcagggcgtaaactttcat	agtaacttttgccctcttgt
tggtgacgcaactggataga	catccatgagcacataacgt	aaagcattgtctgcatagca
gaaaggcacatttggttgca	accttcaaggtaggtgttag	tccaactttctaagcatagt
tggctttaacaaaatcgccc	tttacagcatctacaccaca	aatgttgttgagtgcatcat
agtaaccacaagtagtggca	ccaaagcaccaataggttga	aaccatctcttgcattgttg
tccaaaggcaatagtgcgac	taccaccagctactatagat	gaggtattatgttcaaggga
cctgtatggttacaacctat	gcaaggcatgttactacgat	atagtctggtatgacaacca
cattaagaccttcggaacct	cagcgcagcttcttcaaaag	aaatgttgtaccatcacacg
actgaacaacaccacctgta	tagcacatcactacgcaact	gatttcccacaatgctgatg
tcaaggacgggtttgagttt	ctgaagtcattgagagcctt	tatctgcatctacaacctgt
accttccttaaacttctctt	atagaggtttgtggtggttg	ttaggtgaattgtccatact
gaatgtctgaacactctcct	gatgggaatgccatttttct	gctgttacaataagaggcca
acttctgtgggaagtgtttc	ttacttgtaccatacaaccc	atttgacagcagaattggcc
acagcttcactagtaggttg	gccaaagaccgttaagtgta	gcaacaggactaagctcatt
acaaactggtgtaccaacca	gatcacatgtcttggacagt	agcacaagacatctgtcgta
atcatattaggtgcaagggc	agggttaagcatgtcttcag	caagcagtttgtgtagtacc
aagtaacctttgttggtgca	taggattggctgtatcaacc	agctaacgcattgtcatcag
gtcttgttgaccaacagttt	aacaccagatggtgaaccat	ctccctttgttgtgttgtag
cttatttaaggctcctgcaa	ggtaattccatatggtgcat	cggataacagtgcaagtaca
cacagcagttaaaacaccct	cctgtcaacaaaaggtccat	gcccatttcaaatcctgtaa
ttggcacttttctcaaagct	actgtaatagttgtgtccgt	tccatcactcttagggaatc
tacatagccaagtggcattg	ataacagcagcgtacaacca	ggttccagttctgtatagat
ctagttgtgtagattgtcca	gattgagaaaccacctgtct	cactttaggacctttaggtg
acacttttatcacctctctt	acttcatagccacaaggtta	ccatacctctatttaggttg
tctaggtggaatgtggtagg	gtcttgtgttagaggttcat	gtggcagctaaactaccaag
atgtcattgacatgtccaca	gtttgagcagaaagaggtcc	attaccagcttgtagacgta
gctccatccaaataagttgg	tacgtccattcataccattt	tacagttgaattggcaggca
gtggtagtactcaaaagcct	agtaacacctgagcattgtc	tgtaagctttagcagcatct
gggtatttccactttttagt	ttcttttcactgcactttgg	aattagtgattggttgtccc
cttgtagagcaggtggatta	gtaacaaccagtggtgtgta	ccagtgtgtgtacacaacat

ccacaagttttacacaccac	ccaactagcaggcatataga	gtgtaactgttattgcctga
accacactggtaattaccag	gtatcaaccatatccaacca	ttcttgatccatattggctt
gtaaagcaccgtctatgcaa	agcaccatcatcatacacag	aacacgatgcaccaccaaag
cttgcgtttggatatggttg	ttcataagtgtccacactct	tatgtggcaacggcagtaca
gtatacaccaggtatttggt	aagagcccacatggaaatgg	atcctttaggatttggatga
ctggttttagatcttcgcag	cctctggccaaaaacatgac	ggtatttgtacatacttacc
aagcagcggttgagtagatt	agtcagtctaaagtagcggt	cagggtcattagcacaagtt
acaaccgtctacaacatgca	catctatgctattcttgggt	agacggtacagactgtgttt
tacattcgactcttgttgct	actgtacagtggctactttg	agccataacctttccacata
ctttacctccattagcatag	ttctactctgagttgttgca	ttcgcggagttgatcacaac
gtactaccagcacagaatgt	cacattgagcccacaattta	tgtgcatcagctgactgaag
agtgacaagtctctcgcaac	ttgtttatgtctacagcacc	accgcaaacccgtttaaaaa

**SARS-CoV-2 #4**	**SARS-CoV-2 #5**	**SARS-CoV-2 #6**
aattgtcatcttcgtccttt	cagggggtaattgagttctg	gaaggagtagcatccttgat
catgtttagcaacagctgga	gacagggttatcaaacctct	gttgcagtagcgcgaacaaa
ttcacctaagttggcgtata	accaaaaatccagcctctta	gaaagggagtgaggcttgta
caataccagcatttcgcatg	ataagtagggactgggtctt	gtgcaacgccaacaataagc
gctttgttaagtcagtgtca	actctgaactcactttccat	tgattttggaagcgctctga
tctcttccgtgaagtcatat	ttccttcaaggtccataaga	tagttgccatctctttttga
atttgggtggtatgtctgat	tgagggagatcacgcactaa	aagtgaacacccttggagag
caacacctagctctctgaag	acctattggcaaatctacca	ccagcagcaacgagcaaaag
agaaacccttagacacagca	acccacataataagctgcag	gaacggcatttccagcaaag
cattaatacagccaccatcg	ctttgtttctgagagagggt	aataggacttgttgtgccat
ggttgttgacgatgacttgg	agattctgttggttggactc	cccatttttcagtataacca
gcatcttgatcctcataact	tgcaaatctggtggcgttaa	acacagtcttttactccaga
ccatagaatttgcttgttcc	atttgtctgacttcatcacc	tgagttgagtacagctggta
acttgagcacactcattagc	gcctgatagatttcagttga	ttcaacaccagtgtctgtac
cactagaccttgagatgcat	accattacaaggtgtgctac	atgttcttcaggctcatcaa
cccaataccttgaagtgtta	aacacctgtgcctgttaaac	acaaaggcacgctagtagtc
aagactgtatgcggtgtgta	acagcaacctggttagaagt	aagttcgtactcatcagctt
aagaacacaagccccaacag	atagcaacagggacttctgt	gatggctagtgtaactagca
cctggagcattgcaaacata	acgccaagtaggagtaagtt	gcagtacgcacacaatcgaa
atttgtccagtcacatgttg	gagttgttgacatgttcagc	atagtaccgttggaatctgc
gtctttcagtacaggtgtta	taactagcgcatatacctgc	ggagctttttaagctcttca
tgagcgtttctgctgcaaaa	attttctgcaccaagtgaca	cctattactaggttccattg
agacagcacttcacgtacag	gtgggtatggcaatagagtt	aaaacctattcctgttggca
tttcaaaggtgtactctcct	agattgctgcattcagttga	caagctaaagttactggcca

tctggcattgacaacactca	tcttgttcaacagctattcc	caccggtgatccaatttatt
acatgtctggacctatagtt	cttgcttggttttgatggat	caagccattgcgatagcaat
gttggtagtcccaaaatctt	gcacaaatgaggtctctagc	gaatgaccacatggaacgcg
agcttgtaaagttgccacat	tcatctgtgagcaaaggtgg	cagaatagtgccatggagtg
agtgtcaacactgaggtgtg	gtgcagaagtgtattgagca	ccgattacgagttcactttc
tatgccaggtatgtcaacac	aaccagaagtgattgtaccc	atgtccacgaaggatcacag
gcacgtacatgtcttatagc	tctgtgtaactccaatacca	gttcgtgatgtagcaacagt
gtaggtacagcaactaggtt	gtgagtcttgaattttgcca	tgcagcaaaacctgagtcac
gcactacattccaaggaagt	accacatcttgaagttttcc	ttatagttgccaatcctgta
ctcaggtcctattttcacaa	aactttgaagtctgcctgtg	ctactggaatggtctgtgtt
cagtggaaaagcatgtggca	tctgatttctgcagctctaa	catctgttgtcacttactgt
tgcattaccatggacttgac	tccaagtacacactctgaca	gttgtacctctaacacactc
ttagggttaccaatgtcgtg	ttttcttgtgcagggacata	ccagaagagcaaggttcttt
ctacatcagcttgaggtaca	aaatggcaggagcagttgtg	tgaaatggtgaattgccctc
cttgttttccatgagactca	gacaccttcacgaggaaagt	tgatagacgtgttttacgcc
cgtagcagactttagtggta	catcacagttaccagacaca	gtgaaactgatctggcacgt
cccaaagctcaaatgctaca	ctgtgttgttgacaattcct	tgcagttcaagtgagaacca
tactggtttaatgttgcgct	gcattaatgccagagatgtc	cgtttaggcgtgacaagttt
atcgtttcagttggtttctt	tcattgaggcggtcaatttc	atgcagctacagttgtgatg
ttgaccatcaactctaccat	ccaagttcttggagatcgat	gaataggacacgggtcatca
tttttacggcttctccaatt	tggccattttatatactgct	aaggtgctgattttctagct
gccttctaatttataccgtt	agccagctataaaacctagc	tgatttagaaccagcctcat
cgtttagctagtccaatcag	attgtcaccattactatggc	ggttcctggcaattaattgt
tcatcattatgcctttaggt	ctcagagtcgtcttcatcaa	gcactacaagactacccaat

**SARS-CoV-2 #7**	**ACE2**	**CD147**
tgattttggggtccattatc	agagtgtatgtatgagtgcc	cgctgcggctctataagaa
aacgtaatgcggggtgcatt	agccatgggatcacaacaac	acgagcgagaagcccagag
agttgaatctgagggtccac	gtggaagtaagaaagcctcc	cagtggtgccttgaggaag
gttctccattctggttactg	gttgggcgctattcaaaact	ctcacagtgcaggaccacg
cgacgttgttttgatcgcgc	tcctcatgagattttctctc	gagcagctgtcgtttggac
agacgcagtattattgggta	caggaagagcttgacatcgt	ggtaggtggcatggatgtg
tgttgagtgagagcggtgaa	gcagcagttacagcaacaag	atccacagacagtgtgctg
gggaatttaaggtcttcctt	tccaaaaatgtcttggcctg	tcaaggaccaccacgcttg
aattggaacgccttgtcctc	tgatagaacaggtcttcggc	cgcggatggaggttatgat
catctggactgctattggtg	aaaaggcagaccatttgtcc	gtaaggtgtgtcttggagc
cttcggtagtagccaatttg	tgtagtggatacatttgggc	ccactgctgttcaaagagc
accacgaattcgtctggtag	gcttgactgtgagattctga	gtggccaacgatgtcgacg

gatctttcattttaccgtca	gcctctcattgtagtctaaa	cagacagtgtgtcctcctg
tttgttagcaccatagggaa	ctcttcatataatggcctca	gtgtactgcatctgcaagt
cagttgcaacccatatgatg	aatgatttgctcttgccatc	cagagcggtcatccatgtc
gattgcagcattgttagcag	catagtctcctctccaataa	gaagatgcaggcatagtcc
tagaagccttttggcaatgt	ggtatgttccacatcttcaa	aatgctctgacttcttccc
acgagaagaggcttgactgc	cctcacataggcatgaagat	acgttttctccctcactgg
tgttgcgactacgtgatgag	ccaaaatctaccccacatat	agacttgcagaccagcctc
gcctggagttgaatttcttg	ttctgtccaaagggaactgt	caggggggtgagatgaatc
caggagaagttcccctactg	ccttgaatattctctgtgca	aaccaggaccactcagtga
cagcaaagcaagagcagcat	agcatggaattttcccagaa	cccagagtcagaggtctta
agctggttcaatctgtcaag	ctttctgaacatttcctggg	agagctgttgttgatcacc
tttaccagacattttgctct	cctttgtgcacataaggatc	gctgtggatatcacgatgt
tagtggcagtacgtttttgc	tgatgagctgtcaggaagtc	accaggatcgctgtttatg
gcttgtgttacattgtatgc	atatcatactggatatgccc	agttggtggcgttacacac
tttgttctggaccacgtctg	agcagaaaaggttgtgcagc	tcattttctcctggacact
tggtccccaaaatttccttg	catggaatccttcattagct	ctagacggcttcgcacacg
agttccttgtctgattagtt	ttaggtgtggctgcagaaag	tagtgaccaacaccaggac
caatttgcggccaatgtttg	gggaatttcccctttaaaga	ttccgtcgcttctcataaa
gaagcgctgggggcaaattg	cagtatgtttcatcatgggg	tccttgtcgttcatgtgat
atgcgcgacattccgaagaa	acatggaacagagatgcggg	gagtggaggcagatgctgg
cgaaggtgtgacttccatgc	attggtaaagggtccttgtg	ggtaggcttgggttaggat
ctgtgtaggtcaaccacgtt	ctgcttgacaaagtgcttct	aggttgggacaggaggttg
atttggatctttgtcatcca	atgtcacatttgtgcagagg	caggtgggcttacagcaag
ttgtatgcgtcaatatgctt	cagggttctgattttccaag	agaaaggggtgttgtgggc
ggtaaggcttgagtttcatc	ggctcaaagtagttgagcag	ctctgcatagaaacctcgt
gaagagtcacagtttgctgt	attcttgttctggtctttca	gatccccaatgaacgggtt
atcatccaaatctgcagcag	catttcattgtcgttccatt	agggcggtaatgacacact
ggattgttgcaattgtttgg	aaattagccactcgcacatc	ctcatggccaaggacaaga
tgtggtctgcatgagtttag	aactctaggctgttgtcatt	caagtcagagcctgagctg
acgtttatatagcccatctg	tcagccatatggaaacaggg	catacaggggagcaggtgg
tctacttgtgctatgtagtt	agatcaggatgacaatgcca	atggtggggattggcagtc
	gaggcataaggattttctcc	aggtctttatttgtggggt
	caatgaagatgctctctcct	
	ccaaatacacactttcccta	
	cactgtaggcaaatcaccat	
	aaagcaatgtcctctacctg	

**GRP78**	**TF**	**PLAT**

caccatagggaacttcatct	cctgcgagggaaaaaggagt	tgagctgttcaagccaattt
ttcttgaacacaccaacgca	ctgcgcagtgattcaacaac	agcagtacacacaacagctc
tgatcgttggctatgatctc	caaggaggcagccgagaaag	acggtgaaaagcactccgaa
ctgaagggtcattccaagtg	ttaaacgctttctgtggtgt	cgattgcttctaagcatggg
caagaacttgatgtcctgct	gtttccactccaaaattgtc	ctcactgcaacttttgacgg
tttcttttcaaccaccttga	taggcataatccatgggttt	gacatataccccattgaag
ccacctccaatatcaacttg	atccagagcttatctcaaca	actggcagacaaagtcggag
tttcttctggggcaaatgtt	gaagcatttgattttccatt	ttcccaacaaatccatcagg
tctttcccaaatatgcttca	cacactcagtgtctgtgatt	tctggtatctatgtcacagc
cggcacagtaacaactgcat	tgataagtcaggtgcacgtc	cttgcgttgtaaggcttcag
gctgggcatcattgaagtaa	tgggacagataggacccttg	agtaattatgattccccagg
atgatccgcatgacattcag	aaatggtggttcctccaaat	tgccttaaagacatagcacc
cccaggtcaaaaacgaggat	gtaaaaaatctggggcgttt	tgcagaactctggggtatac
agagacacatcgaaggttcc	ccgagttttgtgtctcggta	acttcttcccttaggacaag
caccacttcaaagacaccat	aaaatgctggattacaggct	cttttccaacatagcagtcc
acagcttgatgaagtgttcc	acattcagttccttgccaac	tgggtaccacgataggttaa
gtcttttctaacgtctttcc	tgttggtgagtcttctactg	tatcagggtcatggaattcc
tcacgacgaagtttctgcac	aaaacatcccggagggtgag	tccatgctgtgtaactcttg
ctcaattcttgcttgatgct	tgtataactcaagtccttgc	ccggcaataattatgtctgc
gtccatgttcaactcttcaa	cttgagcttgactctttctg	ttccattcttcatcacatgg
gcttcatggtagatcggaac	ttagtgtttgtattggctgg	gaaactgcggctgtttgtat
cagagtcttccaacactttc	tgttccaaagggatggtgaa	tgatgtccgtgaaaagtcct
acccaccgacaagaacaatt	agtgaagcagtagcctttag	ctcctgttcttgacgaagat
agctgctgaatcttgggaat	tggagcgaatcacagcttgt	cagcaggaattgatcagcac
tccttgccattgaagaactc	gagctctgctggttatcttt	tttaagatcctggggaggag
atcttgatcaccagagagga	tgagcactccatgttgcttt	tgtcgtaagtgtcatcatca
catcaagcagtaccagatca	ccattatactatcccattca	tcagctgcagtaatgcgatg
ccactgtttcaataccaagt	cccacgatgatgagtgtttc	cttcagccgatcagagaaga
acagtgttccttggaatcag	atgatgaccacgaacaccac	gaagtacagcgactcgatgg
gatctgagacttcttggtgg	gcacttgcacaaatatacgg	gcatattgtttgtgacggtt
ggctgattatcagaagctgt	tgagcgaagtggggagtttt	atgcatcatggacatcttgg
caccttcatagaccttgatt	tgcggtagtgcagtatctag	tagtgacctttgtgtatacc
aaggtgacttcaatctgggg	agaggtcttagtatcagtgc	tgtcttgaatccagtctagg
gctgtcactcgaagaatacc	cgggatcgtcaagctctgtg	tttcttggtcattgcttcat
tcaggcgattttggtcattg	ccacaaagggaacctttctg	ggtgtgtcttctactgaaga
accatcctttcaatttcttc	agagtgttggctgggatatc	tagagtagaggacacttggc
tcagcaaacttctctgcatc	gtgacaaatgctagtgttga	agatacatgctacatcctct
ctctttgagctttttgtctt	gtaccgttcagtacattcta	ccctgaaaacttgcgtcaag

aagcatagctttccaactcg	tgcaaaagacgcggtagtgt	gtgttttctcatcttctcat
ccaatctggttcttgagaga	tagtgtcttcctgagtgtag	aatcctagagttatgggagg
tcaatcttttcctctacagc	tacaaaaggcatctgtcccc	acatgatcttgtgtctcgtg
cacttccatagagtttgcta	gtcaatggaatcaccttctc	tcctgaaagatcaagccgta
ttctgatgtatcctcttcac	agctatcagaaaagtcagcc	acgtattcttgtccctaatg
gcagatcagtacacctacaa	ctgaactttcaagagtacct	tcctcctgtaacagctataa
agttcctgagtccaatattt	attgatgtgagagtttgcca	caacatttctgtctcctaga
gttctctcaattttctccta	atcctcaccattgtagttaa	agtatggggctgtgatctag
actctgaggtgaagattcca	agagacagctgtcaaatgtt	aaaggaatgccttccaggag
agacatgtgagcaactgcta		gctgatgctacttcatcaat

**PAI-1**	**THBD**	**vWF**
gagttttcaaaggtgccttg	tccagtttataaatgcgcgg	catctttcccttccaaatag
ttttcccaaagaccagaagc	cgagttagtctctgacatgt	aaatttcaaacccggcagga
gcgggactcttgaagatgta	acagcttagacgtttctagc	cagtcttttctgtgctaagg
ttttactccgaagtcggtgg	gtgtaagctcccaggaaaac	ttaaagtcaccacaaagccc
cctggaccacatgcttaaac	gagaagaagaaccccaagca	tcctaaagtcatcttcagca
accccatagggagagaaaac	cttgactgcaactcggttag	tcacagagagccacgaaagg
atagcatcttggatctgctg	cctggaaaagcgcaaagcac	ggagacacacactccttata
gtcttctcgctgattttgaa	cactgtcattagatgtcctt	gaacattcggagctctgtac
tgctaatctcgttctggttc	aaagggagatgacttcggca	attcttcattgctgcagatc
aggtctctctggacgaagat	tgagttcacactactgtcgt	ctgcatggtctctatgacaa
gatgatgaatctggctctct	actccacaatagcgaagctg	agtgactgagcgagtacaga
tttggcaagcaagtcactga	tcacacattagctccaaacc	cgtagtaacagggacactgg
agaatgaggcgtgtcagctg	gcacaacgacatctgtgcaa	cactcgtggtacaatgcatg
gttgaagtagagggcgttca	aaatgttcgcagaggtcagt	tgttgcagtcaatcttcact
ccatcagacttgtggaagag	ctcaaagccatcatagcaga	acggagacactgaggtgatg
catcatgggcacagagacag	ttatttccgaagcacggatc	tagtgacagctttcttgtgt
gtagttgaacttgttggtct	cctcagcacagatgcaaatg	cattgtctgcttcatgatgt
catcgggagtggtgaattca	gcagaacaattcacacctgt	agatgtcgctggtaaggatt
tccaggatgtcgtagtagtg	tcaatgtccacgcatatgtg	cacaggagcaagtgtcgtaa
ggagagaggcactttcttat	aagattgcgacactcactgc	tagccactttctcgaacatt
catccaagatgttggtgagg	cacagttgcactcataggag	ggtctgaagaagttcatcca
cctttccattgtttgatgag	ggtcctcgttgtattcaata	gtaaggttgacaccatcaca
gtctccagagagaacttagg	caatggaggtgccaatgaga	aacgtatggggtagtagagc
tatgctgaacatgtccgtca	atagaagcaccaaaagcgcc	aatgcgcttctgagagatgt
aaagacttgtgaagtcggcc	atccaggaaagacgaaggca	aaggtcaatgtaggcatggg
cacctcgatcttgacttttt	tcagccaagtggcttttaag	acgtaacggaccaaattcct

aatggctgtggaggaagacg	tctccagtcatcagttattg	agaaaggccttgttttcagg
cacaaagaggaagggtcggt	tgcttatcatgaggatggga	gcagaggtagccaattattt
ccatgaagaggattgtctct	gaagaaattgggctctcctg	gaactccttgctcttattga
cttctcctccagaaagagtg	cctcactgtgtatttgtcaa	taacggatctctcgcacatg
gaggctggttggaagatttg	caaggtttgcctaaaggcta	atgtagaccagattaggtgc
ctacagtctgggttagacag	tgtggaggctgggaaaaacc	tcggatgaagatgggagcat
gtgtgactggaacacgagtg	cttgtctggagaggaggata	ctcggggaagtgtctcaaag
agataaggcagcgtttcttc	tgtttagctgtggcatttta	catcgatggtattgatgcgt
ccatactgggcaaacacttg	ctgtgacattccagcattat	ctggagttttaccacattgg
acctacactctgaaatagcc	taggactaaggctcatctca	agggtaattatggtggggag
gtgtgccacggcttaaataa	atggcaatgtcctattgcaa	ttactgtgtggcattgatct
ccctgagatacagggatata	actttgctggaagatgcttc	accgtgcttaatctcaatgg
attggggtaagggtgtgtta	aagtcagctaggcacaaagc	atagatgctgacttccatgt
ggtccctaaggaaaaaagcc	gaactattagtggcaggagc	gaacctgacctcatacatga
aatttgaaaccactttgccc	caaagcagcacactgctttt	aatgtgaggatgtggccaag
cattgaagtgactcaccagt	aagacaagaccctaaccagg	accgtacatcttcgaagcaa
acatacagcagccggaaatg	tagatattgtccaagagcct	ctgtcagagacaggacactg
ctcttgcgcaatttttcagt	tcttgtcaacaggctaacgg	cagcaaacagtgctgagagg
gcctgctatgagattacatt	taattggcctaacatagcct	gaagcaatcacctcacacac
ggagacgtaatgctgcagag	gttcattcctagcttagtta	ttatataccagggatgctgg
gctgtccagtgcataatgaa	atctgttccttacactgaca	ctctaggaactgatgtcgga
attcaagaacattcgacccc	ataagcaaaggctttgcctg	acactcatccaggaagagtg

Supplementary Figure S1.

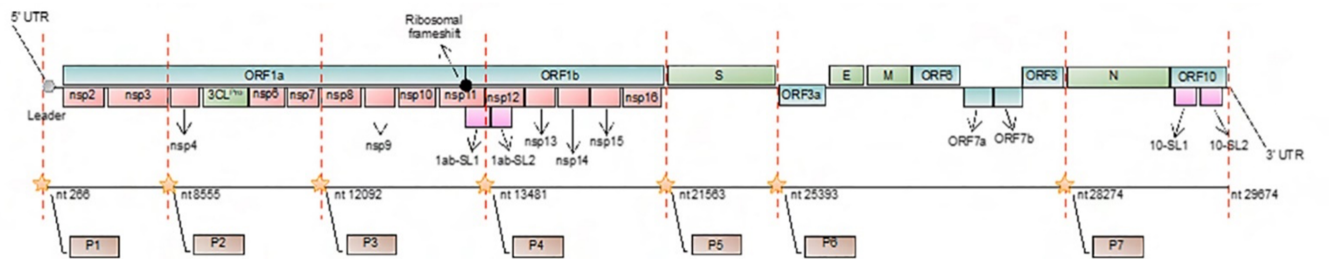

Supplementary Figure S1. Organization and expression of SARS-CoV-2 complete genome and FISH probe diagram targeting its genes. The virus ORFs are defined by the genomic sequence of SARS-CoV-2 isolate Wuhan-Hu-1(NCBI Reference Sequence: NC_045512.2). The ORFs are depicted in blue (ORF1ab, ORF3a, ORF6, ORF7ab, ORF8 and ORF10), non-structural proteins in orange (nsp2-3, nsp 6-11, nsp12 (RNA-dependent RNA polymerase; NiRAN and RdRp), nsp 13 (helicase), nsp14 (3′ → 5′ exonuclease), nsp15 (endoRNAse) and nsp16 (2′-O-ribose methyltransferase)), structural proteins in green (3C-like proteinase, spike (S), envelope (E), membrane (M) and nucleocapsid (N) proteins) as well as frameshifting stimulation elements in pink (ORF1ab – SL1, SL2, and ORF10 – SL1, SL2). The open grey box represents the common 5′ leader sequence and the black circle represents another programmed frameshifting element in the ORF1 fragment. P1 – P7 represent probe set designs with each set containing 43-48 probes (details in Supplementary Table 2.) targeting specific regions of SARS-CoV-2 genes.

Supplementary Figure S2.

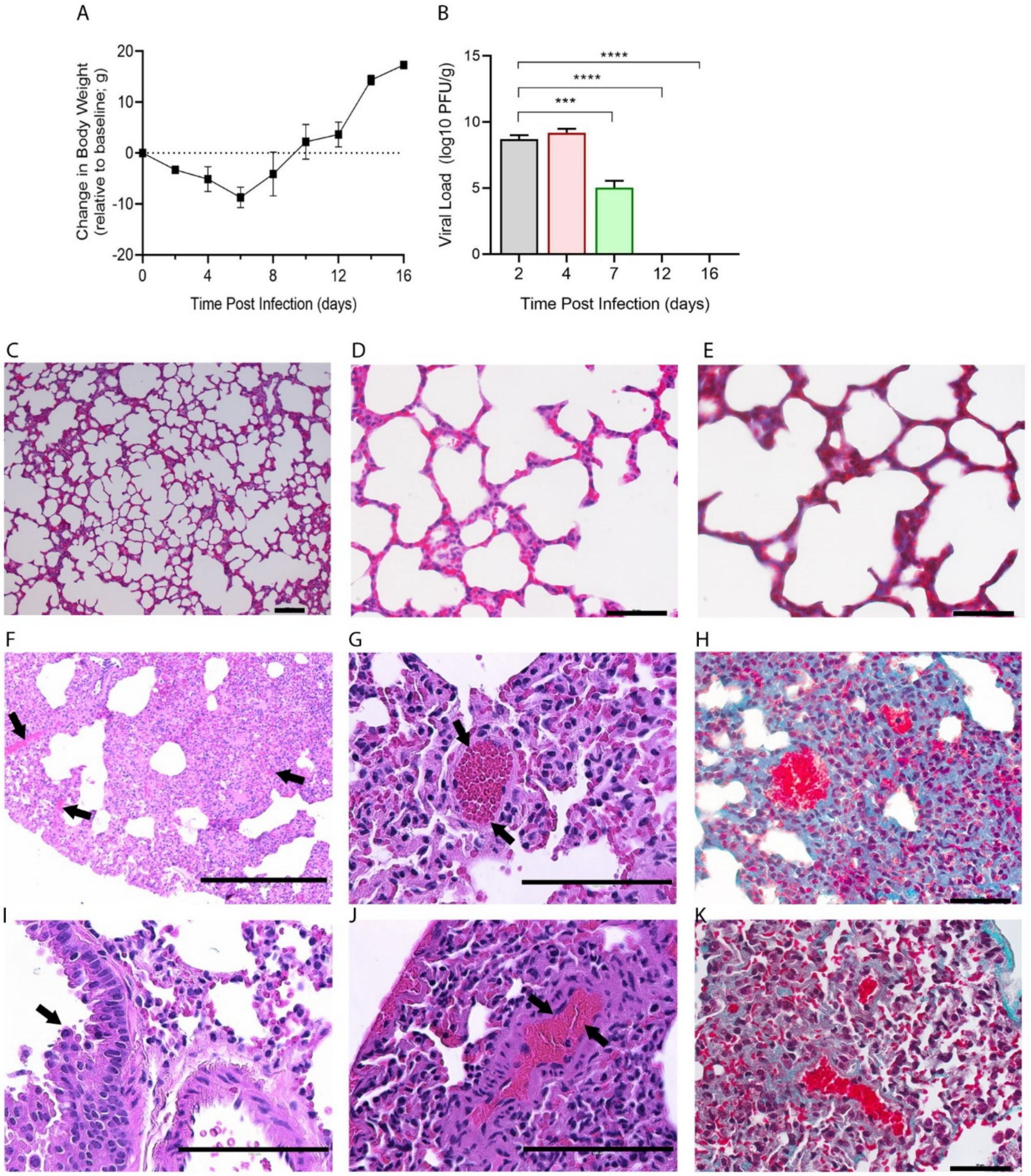

Supplementary Figure S2. Clinical pathology of SARS-CoV-2 infected hamsters. A. Body weight changes in SARS-CoV-2 infected hamsters up to 16 dpi. **B.** Replicative viral load in the lungs as determined by the number of viral plaque-forming units (PFU). No replicative virus was noted at 12 and 16 dpi. Values plotted are mean ± SD (n=3-4 animals per group). Only statistically significant differences were shown in the figure. *** P<0.001 and **** P<0.0001 by unpaired *t*-test. C,D,F,G,I,J. Representative H&E-stained histologic image of lung sections from SARS-coV-2 infected hamsters at 4dpi showing key pathological features related to COVID-19 in humans. E, H, K are representative images of lung sections stained with Masson's Trichrome to visualize tissue remodeling, fibrin and collagen deposition. No clinicopathological signs were noted in the uninfected/control hamster lung sections (C, D, E). SARS-CoV-2 infected hamster lungs show pulmonary occlusion (arrows in F), extravasation of platelets in the pulmonary tissues and adherence of RBCs on the wall of a blood vessel (arrows in G and H), loss of endothelial cells in the vasculature (arrows in I), and red-thrombus tightly

adhered to the walls of pulmonary arterioles and occluded the lumen (arrows inJ and K). Image C is 40x, image D and F are 100x and images E, G-K are 400x. The scale bar represents 100μm in images G, I, J, 400 μm in C, F, 200 μm in D and 50μm in E, H, K.

Supplementary Figure S3.

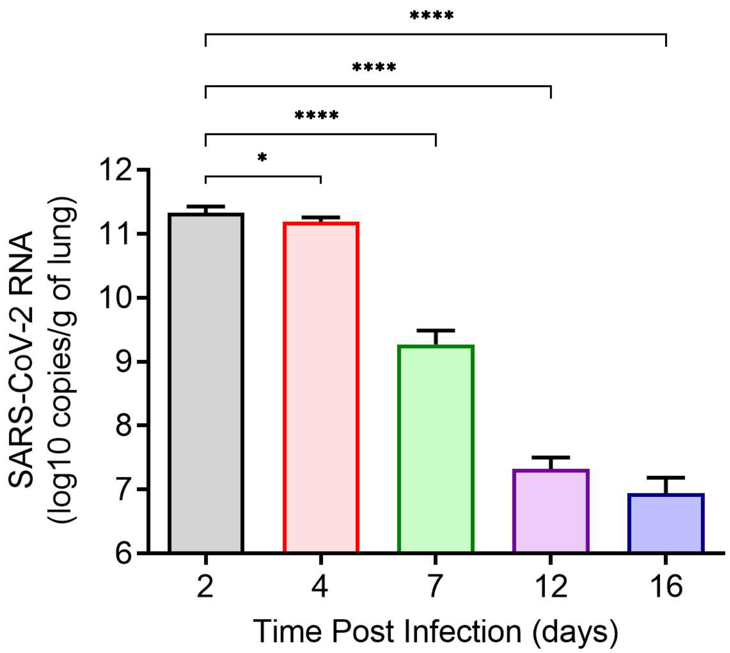

Supplementary Figure S3. Number of total viral RNA copies in the lung of SARS-CoV-2 infected hamsters. Total RNA from lungs harvested over time post-infection was analyzed using SARS-CoV-2 N-specific primers. Values plotted are mean ± SD (n=3-4 animals per group). Only statistically significant differences were shown in the figure. * P<0.01 and ****P<0.0001 by unpaired *t* test.
